# Comparison of host response mechanisms evoked by extended spectrum beta lactamase (ESBL)- and non-ESBL-producing uropathogenic *E. coli*

**DOI:** 10.1186/1471-2180-13-181

**Published:** 2013-08-02

**Authors:** Isak Demirel, Annica Kinnunen, Anna Önnberg, Bo Söderquist, Katarina Persson

**Affiliations:** 1 Faculty of Medicine and Health, Örebro University, Örebro, Sweden; 2 Department of Laboratory Medicine, Clinical Microbiology, Örebro University Hospital, Örebro, Sweden

**Keywords:** Extended spectrum beta-lactamases, Urinary tract infections, Renal epithelial cells, Polymorphonucleated leukocytes, Uropathogenic *E. coli*

## Abstract

**Background:**

Infections caused by extended spectrum beta-lactamases (ESBL)-producing bacteria have been emerging worldwide and the majority of ESBL-producing *E. coli* strains are isolated from patients with urinary tracts infections. The purpose of this study was to compare the host-response mechanisms in human polymorphonucleated leukocytes (PMN) and renal epithelial cells when stimulated by ESBL- or non-ESBL-producing uropathogenic *E. coli* (UPEC) isolates. The host-pathogen interaction of these ESBL-producing strains in the urinary tract is not well studied.

**Results:**

The ability of ESBL strains to evoke ROS-production from PMN cells was significantly higher than that of the non-ESBL strains. The growth of ESBL strains was slightly suppressed in the presence of PMN compared to non-ESBL strains after 30 min and 2 h, but the opposite was observed after 5 and 6 h. The number of migrating PMN was significantly higher in response to ESBL strains compared to non-ESBL strains. Stimulation of A498 cells with ESBL strains elicited lower production of IL-6 and IL-8 compared to non-ESBL strains.

**Conclusion:**

Significant differences in host-response mechanisms were identified when host cells were stimulated by ESBL- or non-ESBL producing strains. The obtained results on the early interactions of ESBL-producing strains with the host immune system may provide valuable information for management of these infections.

## Background

Antibiotic resistance is an emerging health issue that poses a serious threat to public health worldwide. Extended spectrum beta-lactamases (ESBL) are enzymes able to inactivate beta-lactam antibiotics such as penicillins, cephalosporins and monobactams by hydrolysis. ESBL are defined as enzymes that can be transferred, mainly on plasmids, hydrolyse third generation cephalosporins and are inhibited by clavulanic acid, tazobactam or sulbactam [1]. There are three major groups of ESBL enzymes; TEM, SHV and CTX-M and these can be further divided into subgroups. ESBL enzymes are predominantly found in the bacterial species *Klebsiella pneumoniae* and *Escherichia coli* but may also be found in other species of Enterobacteriaceae. These bacteria are common causes of urinary tract infections (UTI) and may also cause sepsis, respiratory tract- and intra-abdominal infections [1]. ESBL-producing organisms have previously been associated with nosocomial infections but community-acquired infections mainly due to CTX-M-producing *E. coli* are emerging [2]. The majority of all ESBL-producing bacteria are isolated from urine samples and most of these bacteria are *E. coli*[3]. Treatment of infections caused by ESBL-producing bacteria is often complicated due to concomitant resistance to other classes of antibiotics such as fluoroquinolones, aminoglycides, trimethoprim/sulfamethoxazole and tetracyclins [4]. The prevalence of ESBL-producing uropathogenic bacteria has increased in the last decades. In southern Europe, 21% of the community [5] and 18% of the nosocomial [6] urinary tract infections (UTI) are caused by ESBL-producing *E. coli*.

The host-responses to infection by uropathogenic *E. coli* (UPEC) are characterized by neutrophil migration into the tissue and production of pro-inflammatory cytokines [7]. The early response of effector cells such as uroepithelial cells and neutrophils to UPEC may influence bacterial clearance and thereby the outcome of the infection. It is not yet established whether ESBL-producing isolates have different virulence properties or pathogenic potentials than non-ESBL producers. Studies performed on expression of virulence factors and phylogenetic groups among ESBL-producing *E. coli* strains have not been conclusive [2,8]. Furthermore, data on the effect of ESBL-producing strains on activation of host effector cells are limited. Some studies have showed that ESBL-producing *K. pneumoniae* are able to impair the respiratory burst of polymorphonuclear leukocytes (PMN) [9] and have a higher ability to invade ileocecal- and bladder epithelium [10] compared to non-ESBL-producing strains. A higher proportion of ESBL-producing *K. pneumoniae* strains were reported to be serum-resistant and therefore able to withstand the bactericidal effect of serum [11]. ESBL-producing *E. coli* have been reported to stimulate higher production of pro-inflammatory cytokines from human monocytes compared to susceptible *E. coli*[12].

It is well known that UPEC can stimulate pro-inflammatory pathways, but recent studies indicate that some UPEC strains have the ability to suppress activation of host-response mechanisms. UPEC were demonstrated to suppress production of pro-inflammatory cytokines from bladder epithelial cells [13,14] and attenuated neutrophil migration [15] compared to non-pathogenic *E .coli* strains. It is not known if ESBL-producing UPEC strains have an enhanced ability to modulate the host-response and evade the immune system or if they are successful in establishing infections only because of their antibiotic resistance. Thus, it remains to be established how ESBL-producing UPEC interact with the host immune system in the urinary tract. The purpose of this study was to compare activation of host-response mechanisms in human PMN and renal epithelial cells when infected by ESBL- or non-ESBL-producing UPEC strains.

## Methods

### Bacterial isolates, cell line and culturing conditions

Eight ESBL-producing and 11 non-ESBL-producing (susceptible) *E. coli*, isolated from standard patient care individuals with suspected pyelonephritis, were obtained from the Department of Microbiology at Örebro University hospital, Sweden. The identity of the patients was anonymized and after that further analyses of the strains were performed. Antimicrobial susceptibility testing was performed as recommended by the Swedish Reference Group for Antibiotics (http://www.srga.org) and the isolates were genetically characterized for CTX-M, TEM and SHV type by real time PCR and nucleotide sequencing and stored as previously described [16]. MG1655, a well-characterized and non-pathogenic *E. coli* K-12 strain and CFT073, a UPEC strain isolated from a patient with pyelonephritis, were used as control strains. The bacteria were cultured on tryptic soy agar (TSA) overnight at 37°C prior to any experiment. Colonies were suspended in phosphate buffered saline (PBS) to the appropriate concentrations.

A498 cells (HTB-44, ATCC) are human renal epithelial cells derived from a kidney carcinoma. A498 cells were cultured in Dulbecco's modified eagle medium (DMEM, Sigma-Aldrich, St. Louis, MO, USA) containing 10% fetal bovine serum (FBS), 1 mM non-essential amino acids, 2 mM L-glutamine, 50 U/ml penicillin and 50 μl/ml streptomycin (all from Invitrogen Ltd, Paisley, UK) at 5% CO_2_ and 37°C. Prior to the experiment the cell-culturing medium was replaced with DMEM containing 2% FBS, 1 mM non-essential amino acids and 2 mM L-glutamine (penicillin and streptomycin were excluded).

### Phylogenetic analysis of *E. coli* strains by real-time PCR

DNA was isolated from 2–3 colonies grown on TSA plates. The colonies were suspended in 100 μl sterile water and the suspensions were boiled for 15 min, cooled to 4°C and subsequently centrifuged for 30 s at 12 000 × g. The amplification was performed by using 10 μl SsoFast EvaGreen® Supermix (Bio-Rad laboratories, CA, USA), 2 μl of primer (250 nM), 2 μl genomic DNA (in total 50 ng) and 6 μl water. All primers were ordered from Eurofins MWG Synthesis GmbH (Ebersberg, Munich, Germany). The amplification was performed in CFX96 Real-time thermocycler (Biorad Laboratories, Hercules, CA, USA) as previously described [17].

### Viability of A498 cells stimulated with *E. coli*

The A498 cell line was stimulated with the different bacterial isolates and the viability of the cells was assessed after 6 h. Multiplicity of infection (MOI) of 10 was used (5 · 10^5^ cells were stimulated with 5 · 10^6^ CFU of bacteria). The viability of the cells was assessed by the trypan blue (0.4%) exclusion test in a cell counter (TC10™ automated cell counter, Bio-Rad) and by the cytotoxicity detection kit plus-LDH (Roche Diagnostics, Indianapolis, IN, USA) according to manufacturer's protocol.

### Isolation of polymorphonucleated leukocytes

Human polymorphonucleated leukocytes (PMN) were isolated from whole blood using polymorphprep (Axis-Shield PoC AS, Oslo, Norway). Blood was collected according to the swedish national board of health and welfares guidelines and the ethical guidelines of the declaration of Helsinki. The healthy volunteers gave a written informed consent for research use and the samples were anonymized immediately after collection. The donors were not subjected to extra harm or risk as the blood was collected at the same occasion as a blood donation. According to paragraph 4 of the swedish law (2003:460) on ethical conduct in human research, this study did not require ethical approval. Briefly, polymorphprep was layered with an equal volume of heparinized blood and centrifuged at 1350 rpm for 40 min at room temperature. The PMN fraction was collected and an equal volume of 0.45% NaCl and 20 ml PBS was added. Any remaining erythrocytes were removed by hypotonic lysis with sterile milliQ water. Cold PBS containing 3.4% NaCl and Krebs-Ringer glucose (KRG) were added to restore osmotic pressure. The PMN were centrifuged, the supernatant discarded and the pellet resuspended in 1 ml PBS, KRG + Ca^2+^ or DMEM + 4-(2-hydroxyethyl)-1-piperazineethanesulfonic acid (HEPES). The viability of the PMN was > 90% as determined by the trypan blue exclusion test.

### Measurement of total ROS-production

Total reactive oxygen species (ROS)-production of PMN was measured with a luminol-horseradish peroxidase (HRP) assay. Luminol is activated by H_2_O_2_ and the evoked luminescence is proportional to ROS-production. PMN in KRG + Ca^2+^ were incubated with luminol (0.1 mg/ml, Sigma) and HRP (4 U/ml, Roche) for 15 min at 5% CO_2_ and 37°C. PMN and bacteria (MOI 10) were combined in a 96-well plate. Phorbol-12-myristat-13-acetat (PMA) was used as positive control and KRG + Ca^2+^ as negative control. The plate was centrifuged at 400 × g at 4°C for 3 min and the luminescence was measured in a microplate reader (Fluostar Optima, BMG Labtech, Aylesbury, UK) every third min for 4 h. All samples were run in duplicate.

### Measurement of bacterial growth

PMN and bacteria (MOI 10) were combined in a 96-well plate in cell culturing medium (DMEM + HEPES). As a control, bacteria were grown in an equal volume of cell culturing medium. The plate was incubated at 5% CO_2_ and 37°C and the absorbance was measured in a microplate reader (Multiska Ascent, Thermo labsystems, Helsingfors, Finland) at 620 nm every 30 min for 6 h. The absorbance of PMN cells only was measured and subtracted from the absorbance of the co-incubated samples (bacteria + PMN). The relative growth inhibition (delta OD620) was calculated as absorbance of bacteria-(absorbance of bacteria + PMN). The viability of the PMN was > 80% as determined by trypan blue exclusion test 6 h after bacterial stimulation.

### Transwell PMN migration assay

A498 cells were seeded onto a inverted 3 μm pore size transwell insert (Falcon, BD Biosciences Pharmingen, San Diego, USA) for 3 h (at 5% CO_2_ and 37°C) to facilitate cell settling. After 3 h the inserts were placed in 6-well plates with fresh medium and the cells were cultured on the inserts for 2 weeks at 5% CO_2_ and 37°C. Medium was changed every second day. The cells were pre-stimulated with the bacteria (MOI 10) for 4 h by adding the different strains to the bottom wells. The PMN were prepared as described above and 10^6^ PMN were added to the top well after the pre-stimulation. PMN cells were collected from the bottom well after 1 and 3 h and counted in a cell counter (TC10™ automated cell counter, Bio-Rad).

### Measurement of epithelial cytokine production

An enzyme-linked immunosorbent assay (ELISA) was performed to measure the cytokine production of A498 cells stimulated with different bacterial strains for 3 and 6 h. The cytokines IL-6 and IL-8 were measured using human IL-8 and IL-6 kits (ELISA MAX™ Deluxe Sets, BioLegend, San Diego, CA, USA).

### Statistical analysis

The variables were normally distributed and differences between groups were evaluated with the unpaired Student’s *t*-test or one-way ANOVA followed by Bonferroni test. Differences were considered statistically significant when p < 0.05. Data were presented as mean ± standard error of the mean (SEM), n = number of independent experiments.

## Results

### Selection and characterization of the UPEC strains

The renal epithelial (A498) cells were stimulated with the different bacterial isolates for 6 h and the cell viability was assessed. Bacterial isolates that decreased the cell viability (> 20%) were not suitable for the *in vitro* infection study design and were excluded. Two ESBL-producing (2/8; 25%) and five non-ESBL-producing (5/11; 45%) isolates were excluded based on this criteria. Six ESBL-producing and six non-ESBL producing isolates remained for investigation. The characteristics of the different isolates included in the study are summarized in Table 1. All ESBL-producing isolates belonged to either the CTX-M-14 or CTX-M-15 enzyme type. The phylogenetic analysis showed that 50% of the susceptible strains belonged to the B2, 33% to the B1 and 17% to the D group. The majority of the ESBL-producing strains belonged to the B2 group (83%) and the rest belonged to the D group (17%) (Table 1).

**Table 1:** Characteristics of the bacterial isolates included in the study

**Isolate**	**ESBL type**	**Phylogenetic group**	**Antibiotic resistance**
ESBL 2	CTX-M-14, TEM-1	B2	CTX, CAZ, CIP, MEC, TZP, TMP
ESBL 3	CTX-M-15, TEM-1	B2	CTX, CAZ, MEC, TZP, TMP
ESBL 5	CTX-M-15	B2	CTX, CAZ, CTB, CIP, TZP, TMP
ESBL 6	CTX-M-14	D	CTX, CAZ, CTB
ESBL 7	CTX-M-15	B2	AmC, CTX, CAZ, CTB, CXM, CIP, SXT
ESBL 8	CTX-M-15	B2	CTX, CAZ, CTB, CIP, MEC, TZP
Susceptible 1	-	B2	TMP
Susceptible 2	-	B2	-
Susceptible 3	-	B1	TMP
Susceptible 4	-	B2	-
Susceptible 7	-	B1	-
Susceptible 11	-	D	-

*CTX* Cefotaxime, *CAZ* Ceftazidime, *CIP* Ciprofloxacin, *MEC*, Mecillinam, *TZP* Pipeacillin/Tazobactam, *TMP* Timetoprim, *CTB* Ceftibuten, *AmC* Amoxicillin + Clavulanic acid, *CXM* Cefuroxim, *SXT* Sulfamethoxazole/Trimetoprim.

### ROS-production of PMN stimulated with ESBL- and non-ESBL-producing *E. coli*

**Figure 1:**
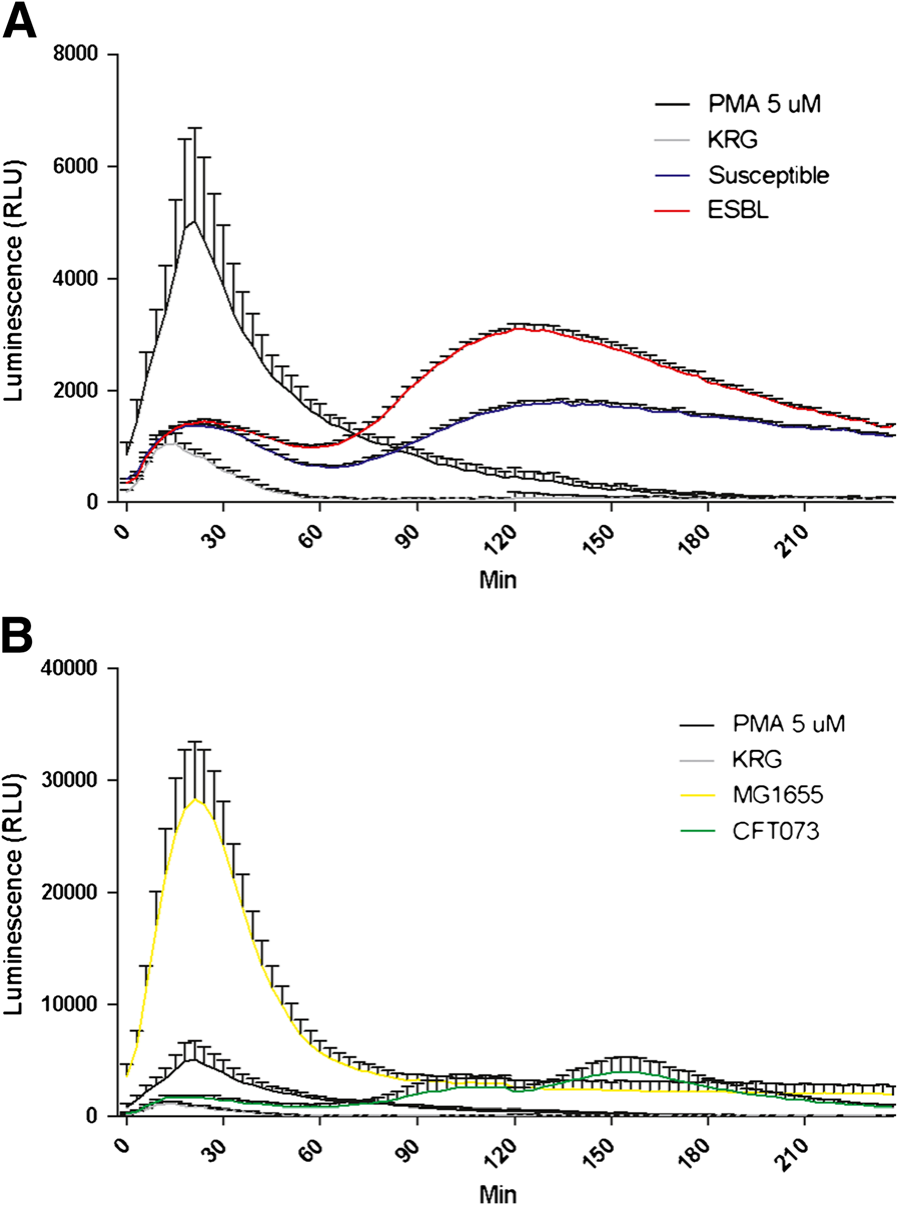
**ROS production induced by ESBL- and non-ESBL-producing *E. coli*.** Total ROS production in PMN stimulated by ESBL-producing strains, susceptible *E. coli* strains, a positive control (PMA) and a negative control (KRG) **(A)**. The ROS production evoked by MG1655, CFT073, a positive control (PMA, 5 μM) and a negative control (KRG) **(B)**. Data are presented as mean ± SEM luminescence (RLU) (n = 4-5 independent experiments).

Production of ROS by PMN is a key characteristic of the early host response to bacterial infections. The ESBL-producing *E. coli* strains evoked higher ROS-production compared to susceptible *E. coli* strains (p < 0.001) when analyzing the sum ROS production for the whole 4 h incubation period. The ROS-production induced by ESBL- producing and susceptible strains followed the same pattern with a low peak after 30 min and a higher peak after 2 h (Figure 1A). The ROS-production of PMN was markedly higher in cells stimulated with the non-pathogenic strain MG1655 compared to those stimulated with the UPEC strain CFT073. MG1655 induced a massive ROS-production after 30 min, approximately 5.5 times higher than the positive control PMA (Figure 1B).

### Growth response of ESBL- and non-ESBL-producing *E. coli* incubated with PMN

**Figure 2:**
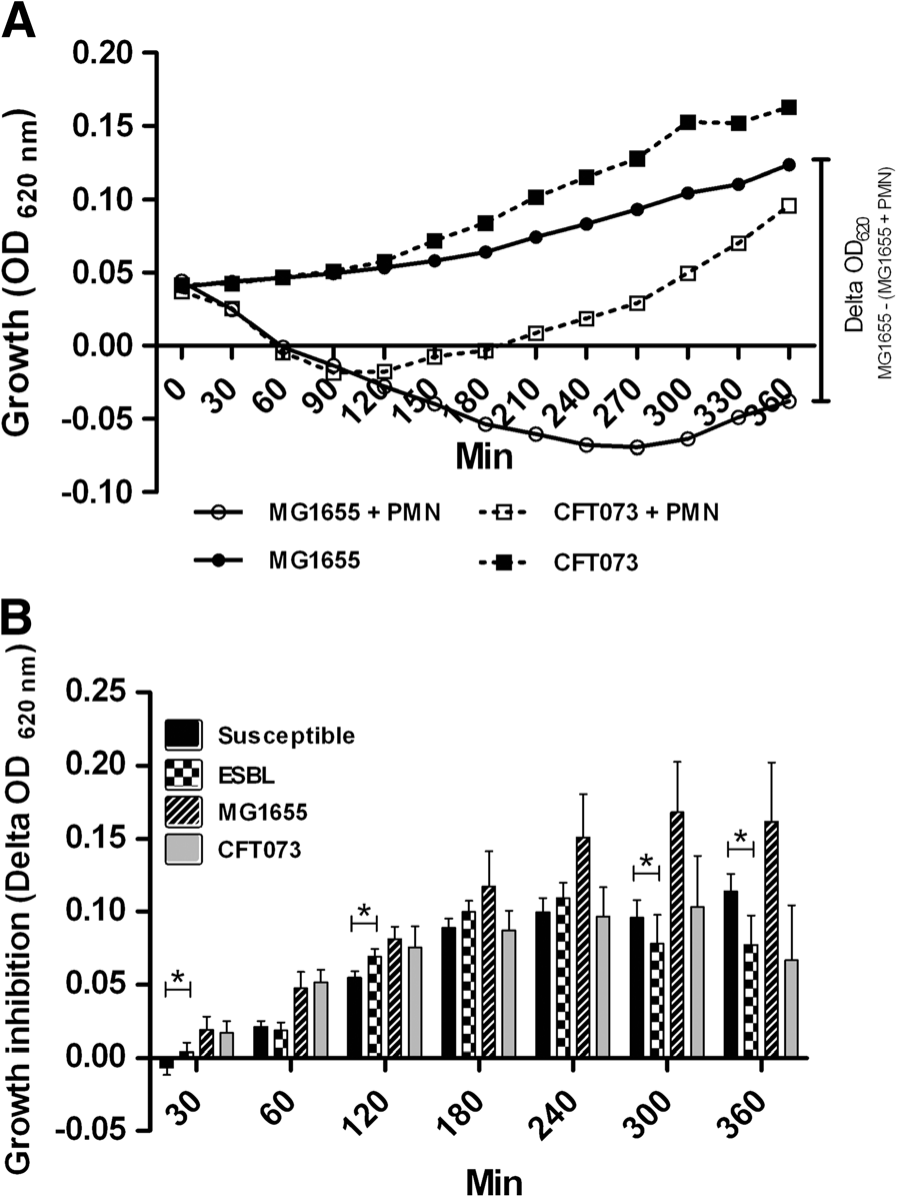
**PMN induced growth inhibition of ESBL- and non-ESBL-producing *E. coli*.** Growth of MG1655 and CFT073 incubated with PMN (MOI 10) or without PMN **(A)**. Relative growth inhibition of MG1655, CFT073 and the mean relative growth inhibition of susceptible and ESBL-producing *E. coli*. The relative growth inhibition (delta OD620) is calculated as (absorbance of bacteria-(absorbance of bacteria + PMN)) **(B)**. Data are presented as mean ± SEM (n = 3 independent experiments). Asterisks denote statistical significance (*p < 0.05).

We next examined whether the observed differences between ESBL- and susceptible strains in evoked ROS production had any effects on the bacterial growth. The bacterial growth response was inhibited in the presence of PMN when compared to bacteria grown in the absence of PMN as shown in Figure 2A. In the presence of PMN, the CFT073 strain showed recovered growth after approximately 100 min while the growth of MG1655 was suppressed for approximately 270 min (Figure 2A). The growth of ESBL-producing *E. coli* was slightly suppressed in the presence of PMN compared to antibiotic susceptible *E. coli* after 30 min and 120 min (p < 0.05) (Figure 2B). However, after 300 and 360 min the growth of susceptible *E. coli* was slightly more suppressed compared to ESBL-producing *E. coli* (p < 0.05). The growth of MG1655 appeared to be more affected by PMN than the other strains but the difference in growth inhibition between MG1655 and CFT073 did not reach statistical significance (Figure 2B).

### Transepithelial migration of PMN evoked by ESBL- and non-ESBL-producing *E. coli*

**Figure 3:**
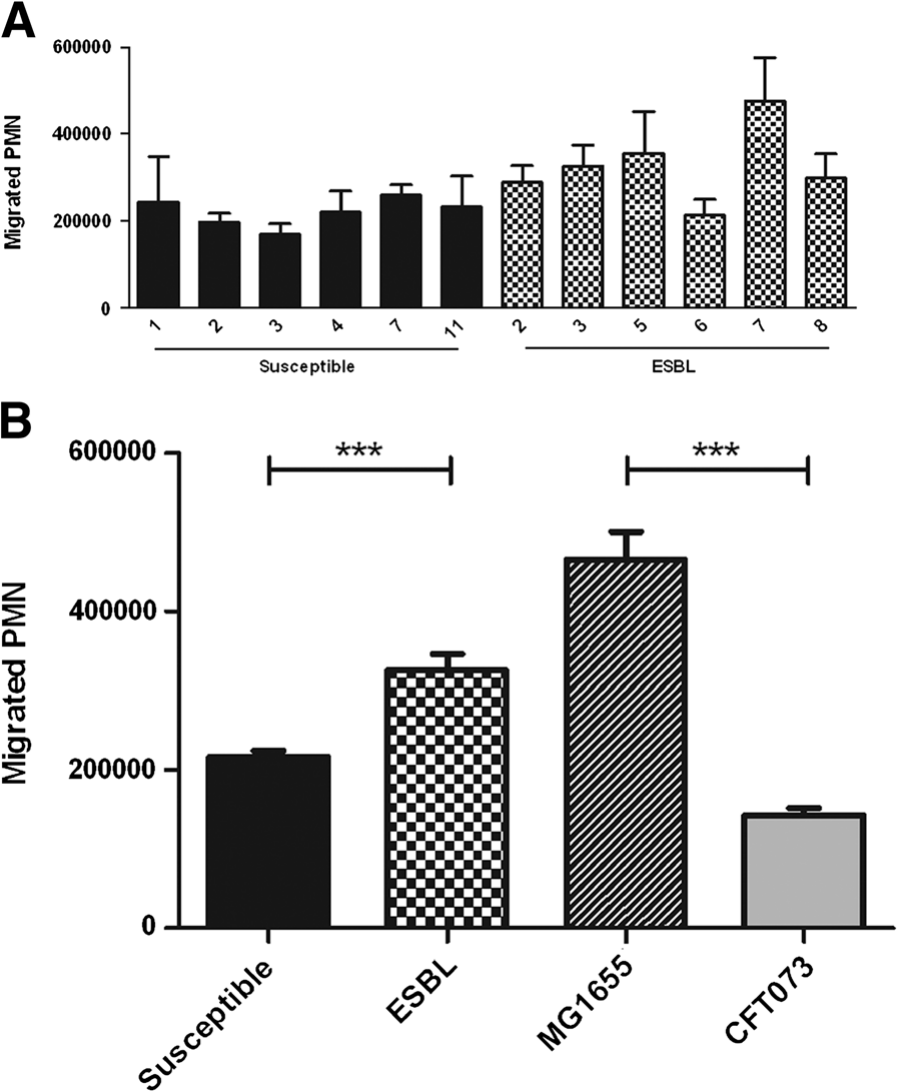
**PMN migration across a renal epithelial cell line layer in response to ESBL- and non-ESBL-producing *E. coli. ***A498 cells stimulated by the individual bacterial strains **(A)**, and the mean PMN migration across A498 cell layer stimulated with ESBL- and non-ESBL-producing strains, CFT073 and MG1655 (MOI 10) **(B).** Data are presented as mean ± SEM (n = 3 independent experiments). Asterisks denote statistical significance (***p < 0.001).

A transepithelial migration assay was performed in order to examine PMN migration evoked by the different *E. coli* strains. The transwell cell monolayer showed low levels of PMN migration in the absence of bacteria (data not shown). All strains evoked PMN migration after 1 h but there were differences in their ability to attract the PMN (Figure 3A). The ESBL-induced PMN migration was significantly higher 1.6 ± 0.13 fold (p < 0.001) than the migration induced by susceptible strains (Figure 3B). The MG1655 strain induced a significant higher 3.3 ± 0.44 fold (p < 0.001) migration than the CFT073 strain. MG1655 was also shown to attract the largest number of PMN compared to the other strains (Figure 3B). There were no differences observed between ESBL-producing and susceptible strains in their ability to attract PMN after 3 h (data not shown).

### Epithelial cytokine production evoked by ESBL- and non-ESBL-producing *E. coli*

**Figure 4:**
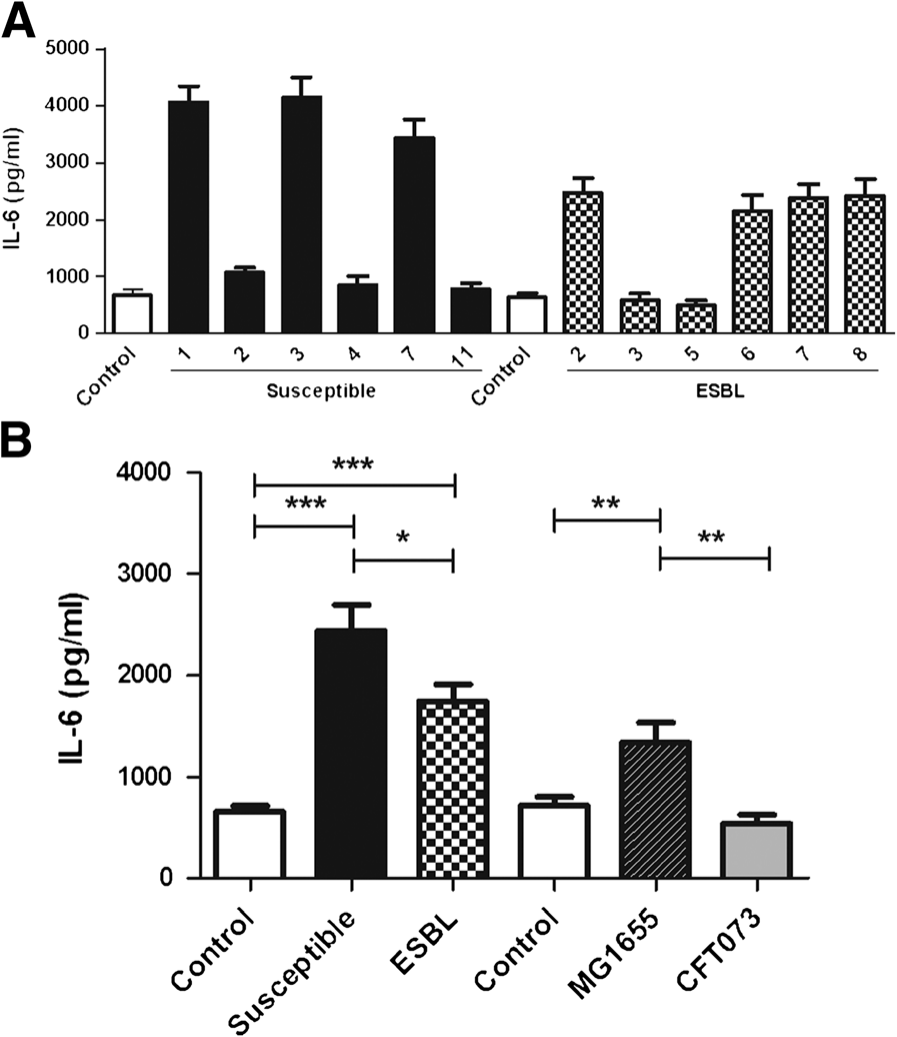
**Induced IL-6 secretion of A498 cells in response to ESBL- and non-ESBL-producing *E. coli*.** IL-6 production from A498 cells induced by the individual bacterial strains **(A)**, and the mean IL-6 production of A498 cells stimulated with ESBL- and non-ESBL-producing strains, CFT073 and MG1655 (MOI 10) **(B)**. Data are presented as mean ± SEM (n = 6 independent experiments). Asterisks denote statistical significance (*p < 0.05, **p < 0.01, ***p < 0.001).

**Figure 5:**
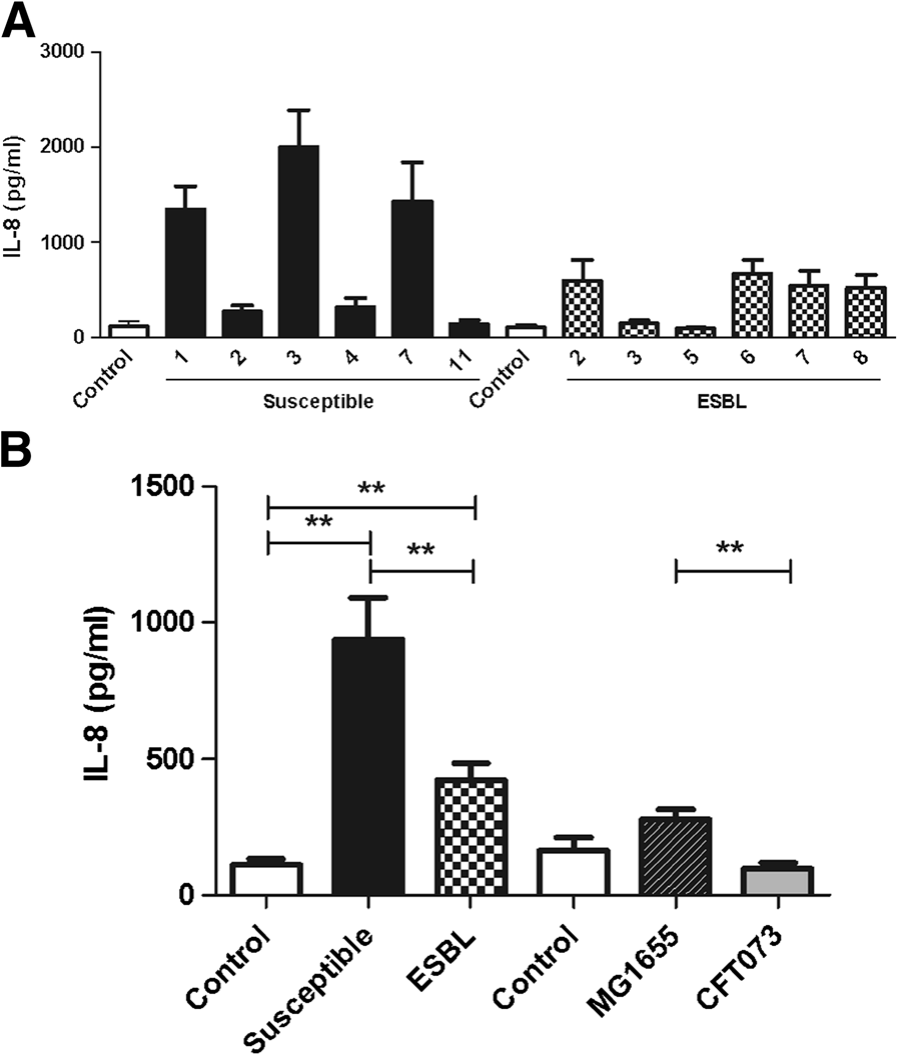
**Induced IL-8 secretion of A498 cells in response to ESBL- and non-ESBL-producing *E. coli*.** IL-8 production from A498 cells induced by the individual bacterial strains **(A)**. The mean IL-8 production from A498 cells stimulated with susceptible and ESBL-producing *E. coli*, CFT073 and MG1655 (MOI 10) **(B)**. Data are presented as mean ± SEM (n = 6 independent experiments). Asterisks denote statistical significance (**p < 0.01).

The activation of pro-inflammatory cytokines from urinary tract epithelial cells was evaluated. Both the ESBL-producing and the susceptible strains induced a significant higher IL-6 and IL-8 production from A498 cells compared to unstimulated cells after 6 h. No significant difference was observed between the ESBL- producing and susceptible strains in their ability to induce cytokine production after 3 h (data not shown). The IL-6 and IL-8 production of A498 cells revealed differences between the individual strains (Figures 4A and 5A) and notably, strains that induced high IL-6 production did also induce high IL-8 production. The cytokine production of A498 cells incubated with ESBL-producing strains when grouped together was significantly lower 28 ± 1.9% (IL-6) and 52 ± 3.5% (IL-8) (p < 0.05) compared to cells stimulated with susceptible strains (Figures 4B and 5B). The non-pathogenic MG1655 strain induced a significantly higher (p < 0.01) IL-6 and IL-8 production than the pathogenic CFT073 strain (Figures 4B and 5B).

## Discussion

In the present study we used an *in vitro* infection model to compare the host response evoked by ESBL-producing strains with non-ESBL-producing strains isolated from patients with pyelonephritis. Two ESBL- producing and five non-ESBL-producing-strains were excluded due to their cytotoxic potential. Thus, the most cytotoxic strains were not included in the study. However, the results suggest that susceptible isolates are more cytotoxic than ESBL isolates at least *in vitro*. Virulence factors such as toxins are known to decrease host cell viability and their expression may partly explain the observed differences in cytotoxicity. Hemolysin, cytotoxic necrotizing factor 1 (CNF1) and secreted autotransporter toxin (sat) have all been shown to be less prevalent in ESBL-producing *E. coli* strains than susceptible isolates [8,18-20].

The ability of ESBL-producing *E. coli* to stimulate oxidative burst and evoke ROS-production from PMN cells was greater than that of the antibiotic susceptible strains. In contrast to our findings, a recent report showed that ESBL-producing *K. pneumoniae* induced lower levels of ROS-production from PMN compared to non-ESBL-producing strains [9]. This indicates that there could be species differences. It has been suggested that one virulence phenotype of UPEC may have the ability to suppress ROS-production from PMN which ultimately could have an advantage in colonizing the urinary tract [15]. Thus, our ROS-production experiments suggest that ESBL-producing strains may be less virulent than the susceptible strains. In support of a negative correlation between ROS activation and virulence, the non-pathogenic strain MG1655 was observed to induce the highest levels of ROS compared to the pathogenic *E. coli* strains.

To compare how ESBL-producing and susceptible UPEC strains respond to the antimicrobial properties of PMN the growth response of the isolates when incubated with PMN was evaluated. In control experiments, the growth of the non-pathogenic MG1655 strain was markedly affected by co-incubation with PMN suggesting that antimicrobial species such as ROS are released in our assay. The fact that MG1655 induced the highest ROS-production of all the examined strains may explain the sustained growth inhibition. Some time-dependent differences in the growth of ESBL-producing and susceptible strains when incubated with PMN were observed. After 30 min and 2 h a slight increase in growth inhibition was observed for the ESBL-producing strains. Interestingly, at these time points ESBL-producing strains induced higher ROS-production from PMN compared to the susceptible strains, which may explain the observed differences in growth inhibition. However, at 5 and 6 h the growth of susceptible strains was slightly reduced compared to ESBL-producing *E. coli*. Thus, it appears that the antimicrobial effect evoked by PMN on ESBL-producing and susceptible strains may vary over time. No differences in the ability of PMN to kill ESBL- and non-ESBL-producing *K. penumoniae* strains were reported in an earlier study [9]. Differences in expression and activity of possible resistance mechanism to antimicrobial factors may also affect the growth outcome. It has been shown that non-pathogenic *E. coli* are more sensitive to ROS exposure, at least in the form of hydroxygen peroxide, than uropathogenic CFT073 [15]. Moreover, UPEC strains have been suggested to secrete effectors that interfere with pro-inflammatory pathways which could decrease the phagocytic activity of PMN cells and partly explain the increased tolerance compared to non-pathogenic strains [15,21,22]. Taken together, the higher evoked ROS production and the trend in growth inhibition of ESBL-producing strains in the early stages of infection may impair or delay the establishment of infection by ESBL-producing strains.

An established *in vitro* transepithelial migration assay with infected A498 cells [23,24] was used to compare PMN migration evoked by ESBL-producing and susceptible *E. coli*, respectively. The results showed that ESBL-producing strains evoked higher PMN migration than the susceptible strains. The non-pathogenic MG1655 strain induced a higher PMN migration than all of the pathogenic strains which has been shown in a previous study [15]. Bacterial suppression of neutrophil migration, mediated by the periplasmatic protein YbcL, has been proposed as an important trait used by uropathogens to modulate host-response pathways [15]. Thus, the higher PMN migration evoked by ESBL-producing strains compared to susceptible strains might impair the propagation and colonization of ESBL strains in the urinary tract. Again, ESBL-producing UPEC strains appear to be less virulent than susceptible UPEC strains based on the suggested association between low ability to suppress neutrophil migration and low virulence [15].

The pro-inflammatory cytokines IL-6 and IL-8 are produced by uroepithelial cells as part of the early immune response and are important for the clearance of the infection [25,26]. Production of IL-6 and IL-8 from renal epithelial cells stimulated with ESBL-producing strains was found to be lower than that of cells stimulated with susceptible strains. In contrast to our results, a recent study found that the IL-6 and IL-8 production of monocytes stimulated by ESBL-producing *E. coli* was higher compared to monocytes stimulated by susceptible *E. coli*[12]. This suggests that ESBL-producing *E. coli* strains have the ability to evoke diverse cytokine patterns from different immunoactive cells. Recent studies have shown that UPEC strains induce lower levels of the pro-inflammatory cytokines IL-6 and IL-8 from bladder epithelial cells than non-pathogenic K-12 strains [13,14] by a mechanisms involving suppressed activation of the pro-inflammatory NF-κB pathway [27]. In our study, the UPEC strain CFT073 evoked minimal cytokine production in support of a suppressive phenotype compared to MG1655 as previously reported [13,14]. The ESBL-producing and susceptible isolates showed variations in their ability to induce IL-6 and IL-8 production. Strains that failed to induce cytokines were found in both groups but notably, among the strains that were able to active cytokines, the cytokine levels were always higher in cells infected by susceptible strains.

A limitation of the present study is that only few isolates were used. However, the included isolates are likely to be representative UPEC isolates as the majority of them belonged to the B2 or D phylogenetic group [8,28]. In a previous study (Önnberg et al., manuscript submitted) the present ESBL-producing *E. coli* isolates were characterized by using rep-PCR (DiversiLab [DL], bioMerieux, Marcy l'Etoile, France). The isolates belonged to three different DL-types and the predominant was DL-type 1 (67%). All DL-type 1 isolates belonged to the ST131 clone. No correlation was found between the ability of the isolates to stimulate ROS or cytokine production with the CTX-M type, phylogenetic group or ST131 clone. Our results are in agreement with previous observations that CTX-M-producing isolates are dominated by the B2 phylogroup and the globally disseminated ST131 clone [29,30]. Further studies are needed to characterize potential virulence factors, including type 1- and P-fimbriae and capsular types among the clinical isolates. The newly identified virulence factor TcpC is of special interest. Some UPEC strains have the ability to secrete effectors like TcpC that are able to suppress innate immune responses, including cytokine secretion from uroepithelial cells [22]. Taken together, if the capacity to suppress cytokine release from uroepithelial cells can be regarded as a virulence characteristic, ESBL-producing UPEC strains appear to be more virulent than susceptible UPEC strains. This is in contrast to the findings from experiments using PMN where ESBL-producing strains appeared to have characteristics supporting low virulence.

Increased knowledge and understanding of bacterial virulence properties may be essential when identifying novel therapeutic targets for multiresistant, ESBL-producing *Enterobacteriaceae*. One virulence property that has been recognized among UPEC strains is their ability to modulate the innate host defense to their favour [13-15]. The majority of the results in the present study strengthens the argument that ESBL-producing *E. coli* strains are less virulent than susceptible strains which has been reported in previous genetic studies [8,28]. ESBL-producing *E. coli* have been reported to express fewer virulence factors than susceptible isolates and CTX-M-producers expressed fewer virulence factors than other types of ESBL-producing *E. coli*[8,28]. In animal models, infection with ESBL-producing *E. coli* showed prolonged survival of the infected animals compared to animals infected with susceptible bacteria [8,12]. The prolonged survival time was correlated to a lower expression of virulence factors [8]. Knowledge of host-bacteria interactions of importance for establishing urinary tract infections by ESBL-producing strains may provide valuable information for improved management of these emerging infections. Targeting bacterial virulence factors is an alternative approach that offers opportunities to inhibit pathogenesis and its consequences without placing immediate life-or-death pressure on the target bacterium [31]. Thus, by inhibiting specific mechanisms that promote infection, *e.g.*, adherens, toxin production, invasion or subversion of host defences, new pharmaceutical tools effective against multiresistant pathogens may be developed.

## Conclusion

In the present study we conclude that differences in evoked host-response mechanisms exist *in vitro* between ESBL-producing and non-ESBL-producing UPEC strains. More research is required to explain the mechanisms behind these differences and also to find out whether differences exist between ESBL-producing and non-ESBL producing UPEC strains in *in vivo* models of UTI.

## Competing interests

The authors declared that they have no competing interests.

## Authors’ contributions

ID and KP design the study. ID, AK, AÖ and BS conducted the experiments. ID, AK, AÖ and KP analyzed the data. ID, AK, BS and KP drafted the article. All authors read and approved the final manuscript.

## References

[B1] Pitout JD Laupland KB Extended-spectrum beta-lactamase-producing Enterobacteriaceae: an emerging public-health concern Lancet Infect Dis 2008 8 3 159 166 10.1016/S1473-3099(08)70041-0 18291338

[B2] Pitout JD Nordmann P Laupland KB Poirel L Emergence of Enterobacteriaceae producing extended-spectrum beta-lactamases (ESBLs) in the community J Antimicrob Chemother 2005 56 1 52 59 10.1093/jac/dki166 15917288

[B3] Khanfar HS Bindayna KM Senok AC Botta GA Extended spectrum beta-lactamases (ESBL) in Escherichia coli and Klebsiella pneumoniae: trends in the hospital and community settings J Infect Dev Ctries 2009 3 4 295 299 19759493 10.3855/jidc.127

[B4] Falagas ME Karageorgopoulos DE Extended-spectrum beta-lactamase-producing organisms J Hosp Infect 2009 73 4 345 354 10.1016/j.jhin.2009.02.021 19596491

[B5] Hoban DJ Lascols C Nicolle LE Badal R Bouchillon S Hackel M Hawser S Antimicrobial susceptibility of Enterobacteriaceae, including molecular characterization of extended-spectrum beta-lactamase-producing species, in urinary tract isolates from hospitalized patients in North America and Europe: results from the SMART study 2009–2010 Diagn Microbiol Infect Dis 2012 74 1 62 67 10.1016/j.diagmicrobio.2012.05.024 22763019

[B6] Yumuk Z Afacan G Nicolas-Chanoine MH Sotto A Lavigne JP Turkey: a further country concerned by community-acquired Escherichia coli clone O25-ST131 producing CTX-M-15 J Antimicrob Chemother 2008 62 2 284 288 10.1093/jac/dkn181 18453527

[B7] Ragnarsdottir B Fischer H Godaly G Gronberg-Hernandez J Gustafsson M Karpman D Lundstedt AC Lutay N Ramisch S Svensson ML TLR- and CXCR1-dependent innate immunity: insights into the genetics of urinary tract infections Eur J Clin Invest 2008 38 Suppl 2 12 20 18826477 10.1111/j.1365-2362.2008.02004.x

[B8] Lavigne JP Blanc-Potard AB Bourg G Moreau J Chanal C Bouziges N O'Callaghan D Sotto A Virulence genotype and nematode-killing properties of extra-intestinal Escherichia coli producing CTX-M beta-lactamases Clin Microbiol Infect 2006 12 12 1199 1206 10.1111/j.1469-0691.2006.01536.x 17121626

[B9] Sahly H Aucken H Benedi VJ Forestier C Fussing V Hansen DS Ofek I Podschun R Sirot D Sandvang D Impairment of respiratory burst in polymorphonuclear leukocytes by extended-spectrum beta-lactamase-producing strains of Klebsiella pneumoniae Eur J Clin Microbiol Infect Dis 2004 23 1 20 26 10.1007/s10096-003-1047-7 14652783

[B10] Sahly H Navon-Venezia S Roesler L Hay A Carmeli Y Podschun R Hennequin C Forestier C Ofek I Extended-spectrum beta-lactamase production is associated with an increase in cell invasion and expression of fimbrial adhesins in Klebsiella pneumoniae Antimicrob Agents Chemother 2008 52 9 3029 3034 10.1128/AAC.00010-08 18573929 PMC2533491

[B11] Sahly H Aucken H Benedi VJ Forestier C Fussing V Hansen DS Ofek I Podschun R Sirot D Tomas JM Increased serum resistance in Klebsiella pneumoniae strains producing extended-spectrum beta-lactamases Antimicrob Agents Chemother 2004 48 9 3477 3482 10.1128/AAC.48.9.3477-3482.2004 15328114 PMC514775

[B12] Bristianou M Panagou C Adamis T Raftogiannis M Antonopoulou A Chrisofos M Galani I Kanellakopoulou K Tsaganos T Giamarellos-Bourboulis EJ The impact of multidrug resistance on the pathogenicity of Escherichia coli: an experimental study Int J Antimicrob Agents 2008 31 3 216 223 10.1016/j.ijantimicag.2007.10.028 18248963

[B13] Billips BK Forrestal SG Rycyk MT Johnson JR Klumpp DJ Schaeffer AJ Modulation of host innate immune response in the bladder by uropathogenic Escherichia coli Infect Immun 2007 75 11 5353 5360 10.1128/IAI.00922-07 17724068 PMC2168307

[B14] Hunstad DA Justice SS Hung CS Lauer SR Hultgren SJ Suppression of bladder epithelial cytokine responses by uropathogenic Escherichia coli Infect Immun 2005 73 7 3999 4006 10.1128/IAI.73.7.3999-4006.2005 15972487 PMC1168571

[B15] Loughman JA Hunstad DA Attenuation of human neutrophil migration and function by uropathogenic bacteria Microbes Infect 2011 13 6 555 565 10.1016/j.micinf.2011.01.017 21315174 PMC3092803

[B16] Onnberg A Molling P Zimmermann J Soderquist B Molecular and phenotypic characterization of Escherichia coli and Klebsiella pneumoniae producing extended-spectrum beta-lactamases with focus on CTX-M in a low-endemic area in Sweden APMIS 2011 119 4–5 287 295 21492229 10.1111/j.1600-0463.2011.02730.x

[B17] Doumith M Day MJ Hope R Wain J Woodford N Improved multiplex PCR strategy for rapid assignment of the four major Escherichia coli phylogenetic groups J Clin Microbiol 2012 50 9 3108 3110 10.1128/JCM.01468-12 22785193 PMC3421818

[B18] Nielubowicz GR Mobley HL Host-pathogen interactions in urinary tract infection Nat Rev Urol 2010 7 8 430 441 10.1038/nrurol.2010.101 20647992

[B19] Vila J Simon K Ruiz J Horcajada JP Velasco M Barranco M Moreno A Mensa J Are quinolone-resistant uropathogenic Escherichia coli less virulent? J Infect Dis 2002 186 7 1039 1042 10.1086/342955 12232848

[B20] Wiles TJ Kulesus RR Mulvey MA Origins and virulence mechanisms of uropathogenic Escherichia coli Exp Mol Pathol 2008 85 1 11 19 10.1016/j.yexmp.2008.03.007 18482721 PMC2595135

[B21] Hofman P Le Negrate G Mograbi B Hofman V Brest P Alliana-Schmid A Flatau G Boquet P Rossi B Escherichia coli cytotoxic necrotizing factor-1 (CNF-1) increases the adherence to epithelia and the oxidative burst of human polymorphonuclear leukocytes but decreases bacteria phagocytosis J Leukoc Biol 2000 68 4 522 528 11037974

[B22] Yadav M Zhang J Fischer H Huang W Lutay N Cirl C Lum J Miethke T Svanborg C Inhibition of TIR domain signaling by TcpC: MyD88-dependent and independent effects on Escherichia coli virulence PLoS Pathog 2010 6 9 e1001120 10.1371/journal.ppat.1001120 20886104 PMC2944809

[B23] Agace WW Patarroyo M Svensson M Carlemalm E Svanborg C Escherichia coli induces transuroepithelial neutrophil migration by an intercellular adhesion molecule-1-dependent mechanism Infect Immun 1995 63 10 4054 4062 7558319 10.1128/iai.63.10.4054-4062.1995 PMC173570

[B24] Godaly G Proudfoot AE Offord RE Svanborg C Agace WW Role of epithelial interleukin-8 (IL-8) and neutrophil IL-8 receptor A in Escherichia coli-induced transuroepithelial neutrophil migration Infect Immun 1997 65 8 3451 3456 9234811 10.1128/iai.65.8.3451-3456.1997 PMC175488

[B25] Hang L Frendeus B Godaly G Svanborg C Interleukin-8 receptor knockout mice have subepithelial neutrophil entrapment and renal scarring following acute pyelonephritis J Infect Dis 2000 182 6 1738 1748 10.1086/317599 11069247

[B26] Uehling DT Johnson DB Hopkins WJ The urinary tract response to entry of pathogens World J Urol 1999 17 6 351 358 10.1007/s003450050160 10654365

[B27] Klumpp DJ Weiser AC Sengupta S Forrestal SG Batler RA Schaeffer AJ Uropathogenic Escherichia coli potentiates type 1 pilus-induced apoptosis by suppressing NF-kappaB Infect Immun 2001 69 11 6689 6695 10.1128/IAI.69.11.6689-6695.2001 11598039 PMC100044

[B28] Deschamps C Clermont O Hipeaux MC Arlet G Denamur E Branger C Multiple acquisitions of CTX-M plasmids in the rare D2 genotype of Escherichia coli provide evidence for convergent evolution Microbiology 2009 155 Pt 5 1656 1668 19359321 10.1099/mic.0.023234-0

[B29] Rogers BA Sidjabat HE Paterson DL Escherichia coli O25b-ST131: a pandemic, multiresistant, community-associated strain J Antimicrob Chemother 2011 66 1 1 14 10.1093/jac/dkq415 21081548

[B30] Karfunkel D Carmeli Y Chmelnitsky I Kotlovsky T Navon-Venezia S The emergence and dissemination of CTX-M-producing Escherichia coli sequence type 131 causing community-onset bacteremia in Israel Eur J Clin Microbiol Infect Dis 2012 32 4 513 521 23117265 10.1007/s10096-012-1765-9

[B31] Cegelski L Marshall GR Eldridge GR Hultgren SJ The biology and future prospects of antivirulence therapies Nat Rev Microbiol 2008 6 1 17 27 10.1038/nrmicro1818 18079741 PMC2211378

